# Evolutionary and Physiological Importance of Hub Proteins

**DOI:** 10.1371/journal.pcbi.0020088

**Published:** 2006-07-14

**Authors:** Nizar N Batada, Laurence D Hurst, Mike Tyers

**Affiliations:** 1 Samuel Lunenfeld Research Institute, Mount Sinai Hospital, Toronto, Canada; 2 Department of Biology and Biochemistry, University of Bath, Bath, United Kingdom; 3 Department of Medical Genetics and Microbiology, University of Toronto, Toronto, Canada; European Molecular Biology Laboratory, Germany

## Abstract

It has been claimed that proteins with more interaction partners (hubs) are both physiologically more important (i.e., less dispensable) and, owing to an assumed high density of binding sites, slow evolving. Not all analyses, however, support these results, probably because of biased and less-than reliable global protein interaction data. Here we provide the first examination of these issues using a comprehensive literature-curated dataset of well-substantiated protein interactions in *Saccharomyces cerevisiae.* Whereas use of less reliable yeast two-hybrid data alone can reject the possibility that local connectivity correlates with measures of dispensability, in higher quality datasets a relatively robust correlation is observed. In contrast, local connectivity does not correlate with the rate of protein evolution even in reliable datasets. This perhaps surprising lack of correlation with evolutionary rate appears in part to arise from the fact that hub proteins do not have a higher density of residues associated with binding. However, hub proteins do have at least one other set of unusual features, namely rapid turnover and regulation, as manifest in high mRNA decay rates and a large number of phosphorylation sites. This, we suggest, is an adaptation to minimize unwanted activation of pathways that might be mediated by adventitious binding to hubs, were they to actively persist longer than required at any given time point. We conclude that hub proteins are more important for cellular growth rate and under tight regulation but are not slow evolving.

## Synopsis

Why do some proteins evolve so very slowly? Why are only a few proteins uniquely vital to the functioning of an organism? Understanding how proteins interact with other proteins may provide the answers. Some proteins are, it is suggested, like hubs on a wheel with multiple spokes (interacting partners) attached: take away a spoke and the wheel works, take away the hub and the wheel is useless. With so many proteins to bind with, hubs may also be as slow evolving as some interaction sites are constrained in their evolution. Unfortunately, prior analyses have been equivocal, not least because of an uncertainty about which proteins interact with which others. Here the authors employ an extensive literature-curated dataset of reliable protein–protein interactions to address the issue of essentiality, connectivity, and evolutionary rate. This study finds that hubs are more likely to be essential, and if not essential, at least have a larger impact on fitness. However, hub proteins are not slow evolving, in part, because hubs do not have a higher density of binding sites. Hub proteins do, however, appear to be under strong regulation, an adaptation the authors suggest that minimizes the risk of unwanted activation.

## Introduction

Protein-interaction networks may be scale-free networks [but see 1]. Unlike random networks in which the number of connections between entities follows a Poisson distribution, in a scale-free network the distribution of the number of connections follows a power law, such that a few members called hubs have very large numbers of connections. The properties of these hub proteins are of particular interest, not least because they may be good targets for antimicrobial agents. How, if at all, are the hub proteins different from other proteins? Intuitively, one might expect many differences. As some classes of protein–protein interaction sites are slow evolving [2], proteins with many partners might be expected to be slower evolving, as claimed [3–6]. Likewise, it seems intuitive that hub proteins may be more likely to be essential (i.e., knockout-inviable), also as claimed [7–10]. Similarly, we might expect there to be a correlation between growth rate of cells lacking a given protein and the number of partners of that protein.

To this list we should like to add a further possibility, namely that to minimize potentially hazardous cross talk, temporal control of the abundance and activity of hubs needs to be regulated tightly to enable continuous equilibration with binding partners and curb excessive flux through certain pathways. As such, we expect the mRNAs might be adapted to be quickly removed when synthesis of the protein is no longer needed, and the hub proteins themselves should be regulated by phosphorylation more than the average protein.

While the above features may appear intuitively reasonable, it is far from clear that any of the prior claims are robust. The main problem is the source of protein–protein interaction data. The large datasets applied to these problems have been derived from high-throughput experiments, which, in the case of protein–protein interactions are known to have both high false-positive [11,12] and high false-negative rates [13]. More generally, use of yeast two-hybrid data often fail to replicate results derived from alternative sources [see e.g., 14–16]. As regards rates of protein evolution, expression level is by far the strongest predictor of rates of evolution [17,18]. There remains debate as to whether, when controlling for rates of expression, the more connected proteins have rates of evolution any different from the average [3,15,19,20]. Similarly, while some studies suggest that protein–protein interaction networks are scale-free and hubs tend more commonly to be the essential parts of the network [7–10], both the scale-free nature of yeast protein–protein interaction network [1] and the relationship between dispensability and position in the network [14] may also be artifacts of biased data.

Here we re-evaluate these issues, taking advantage of a recent effort that assembled a set of 11,334 interactions obtained by systematically curating the past 30 years of Saccharomyces cerevisiae primary literature [13]. This we refer to as the literature-curated protein interaction (LC-PI) dataset. Specifically, we examine three issues: are more highly connected proteins more “important,” do they evolve at lower rates, and are they more tightly regulated? Our premise is that interactions reported in focused primary papers are inherently more reliable than high-throughput interactions, not least because low-scale experiments are done by experts in the field and the validity of interactions are scrutinized by peer review; moreover, multiple contextual information and other self-consistency checks and validations normally support the demonstration of these physical interactions, thereby reducing the false-positive error rate. For any correlation to be real it should then be transparent in this dataset.

### Comparing Literature-Curated and High-Throughput Data

The LC-PI and high-throughput protein interaction (HTP-PI) datasets are in many regards rather different. While, for example, the two are supposed to be measuring the same attribute (i.e., the identity and number of different proteins a given protein interacts with), the correlation between the two sets as regards number of interactants of each protein, although naturally highly significant, is relatively modest (Spearman rank correlation *r* = 0.37). The two datasets also disagree substantially on which proteins are highly connected. Comparing, for example, the top 10% by connectivity in the two datasets we find only 29% in common (see Dataset S1 for LC hubs and Dataset S2 for HTP-PI hubs). Restricting to the top 5%, the figure drops to 24%. Moreover, among the bait proteins common to both sets, ~70% of interactions reported in the literature were absent in HTP-PI [13].

We can also ask if known biases affect both sets equally. Some affinity methods (e.g., tandem affinity purification) will preferentially capture interactions for highly expressed proteins. As these dominate the HTP datasets, it is no surprise that there exists a strong positive correlation between abundance or rate of expression (measured by codon adaptation index [CAI]) and connectivity (abundance: connectivity, *r* = 0.19, *n* = 3,001, *p* < 0.0001; CAI: connectivity, *r* = 0.19, *n* = 4,169, *p* < 0.0001). In the LC dataset, in contrast, this effect is much diminished (abundance: connectivity, *r* = 0.046, *n* = 2,464, *p* = 0.02; CAI: connectivity, *r* = 0.037, *n* = 4,169, *p* = 0.034). Indeed, if we look at essential singleton genes alone, for example, the effect goes away in the LC set (abundance: connectivity, *r* = −0.006, *n* = 185, *p* = 0.93; CAI: connectivity, *r* = −0.002, *n* = 242, *p* = 0.98), while remaining relatively robust in the HTP set (abundance: connectivity, *r* = 0.2, *n* = 204, *p* = 0.004; CAI: connectivity, *r* = 0.12, *n* = 260, *p* = 0.05). As a correlation between abundance and connectivity is an expected artifact of some affinity methods, the weakening of this signal provides some reassurance that the LC dataset is less biased. On an anecdotal level, consider, for example, the chaperone Hsp70, which binds to and facilitates the correct folding of many proteins. The Hsp70 family members Ssa1/Yal005c and Ssa3/Ybl075c have very high connectivity in the LC dataset (44 and 18 interactions, respectively) but not in HTP dataset (two interactions for each).

In other regards, the two networks show similar properties. For example, the LC-PI dataset shows a scale-free connectivity distribution [13], just as HTP datasets do [21–24]. While LC interactions are more likely to be real, we note that there are, nonetheless, inevitable selection biases present in curation data. Although the LC dataset represent over half the genes in yeast, it tends to favor more important or conserved proteins [13], these in general being more closely scrutinized in biology. Given the potentially confounding differences between different datasets, we next examine how robust various conclusions drawn to date from different data sources might be.

## Results

### Highly Connected Proteins Are Less Dispensable

Recently there has been dispute as to whether more highly connected proteins are more likely to be essential. Notably, Coulomb et al. [14] have suggested that prior claims for such a relationship [7–10] may be dataset artifacts. Intuitively, one would expect that loss of a highly connected protein would be more detrimental to the cell than the loss of lowly connected proteins; as in the former case, more processes would be affected than in the later case. Are then highly connected proteins more likely to be essential and for those that are nonessential do the relevant knockout strains show lower growth rates? To address these issues we made use of systematic knockout studies. As previously noted, use of laboratory differential growth rate due to gene-knockout as an estimate of dispensability may not be without faults: many proteins are likely to perform functions that are important in the environment relevant to yeast evolution, but superfluous in the laboratory conditions in which growth rates are measured [25]. With this caveat in mind, we define the importance of a gene by the fitness reduction caused by the deletion of the gene in standard laboratory conditions.

### For Nonessential Genes, Connectivity Correlates with Knockout Growth Rate

**Figure 1:**
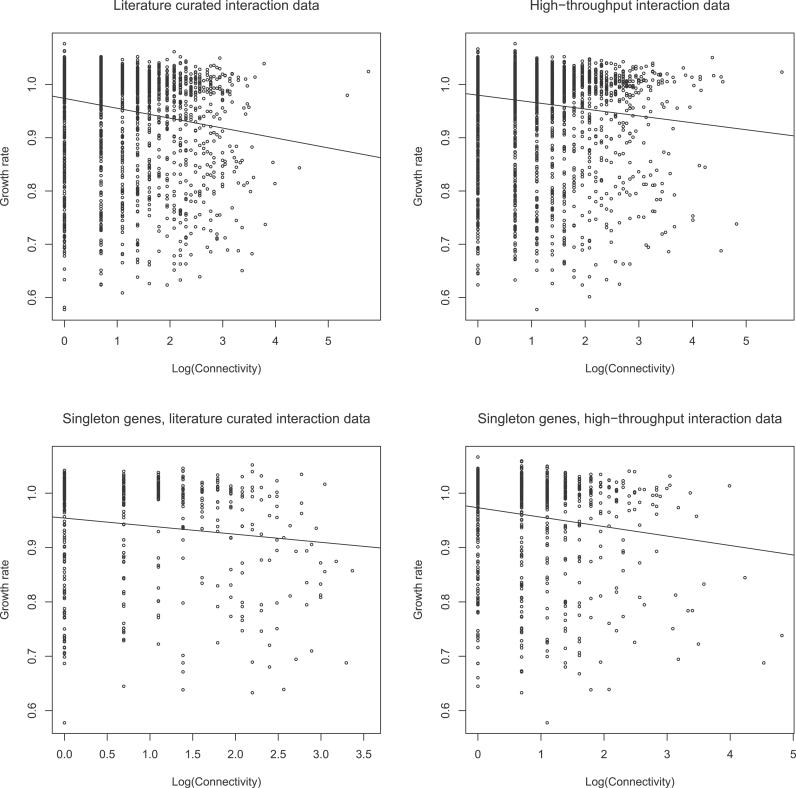
Relationship between Connectivity and Dispensability Scatter plots of growth defect upon homozygous deletion of a gene and the natural log of connectivity of the gene in the interaction network. Growth rate data are from Steinmetz et al. [45].

In both the LC and HTP datasets the more connected a protein the lower the growth rate of the knockout strain, assuming the strain is capable of growth (Figure 1). This effect is also manifest for singleton genes (Figure 1 and Table 1). However, if expression level for the analysis of singleton genes in the LC dataset is accounted for by Spearman partial correlation [26], the connectivity effect becomes non-significant when controlled by protein abundance but not when controlled by CAI [27]. This weakening of the result when employing protein abundance as the controlling variable is not, however, likely to be owing to the effects of the covariate, as among the nonessential genes, connectivity and expression parameters do not significantly correlate in the LC dataset (unpublished data). Instead, this increase in *p-*value appears to be owing to a reduced sample size in the covariate-controlled analyses. Two pieces of evidence support this suggestion. First, the value of *r* remains largely unaffected by covariate control. Second, we performed simulations to mimic the effects of reducing the sample size. If there were *N* data points in the non-covariate controlled analysis and *M* in the covariate controlled tests (i.e., *N* > *M*), we randomly selected *M* from *N,* performed a Spearman rank correlation, and asked how often the observed *p-*value was the same or greater than that observed in the covariate-controlled analysis. In both datasets of growth rate data, we failed to reject the null hypothesis that the increase in *p-*value was owing to a reduction in sample size (unpublished data).

**Table 1:**
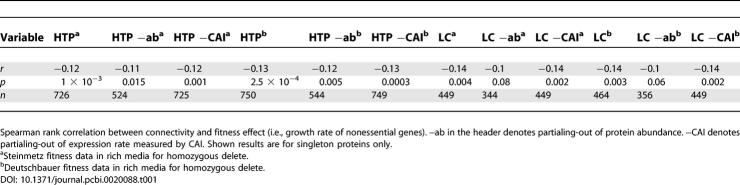
Correlations between Connectivity and Fitness

Might the above results be dataset artifacts? The LC data are, for example, enriched for functionally important proteins that make up the translational machinery. The ribosome and other components of the translational machinery are highly expressed and central for almost all the cellular functions. Removing translation-associated proteins did not change the conclusion (Table S1A) but instead increased the correlation strength. The stronger correlation for non-ribosomal data can be explained because ribosomal proteins have an average connectivity of 4.8 while the mean connectivity of all proteins is approximately 7. Thus, loss of these lowly connected, but functionally important proteins increases the strength of the correlation.

The LC data contain interactions assessed using various different experimental methods [13]; however, affinity purification and two-hybrid based methods account for most of the interactions. These two protocols tend to capture different sorts of proteins. Notably, for both sets of fitness data, the mean fitness of knockouts for proteins in the yeast two-hybrid data were on average higher than for proteins in affinity purification data (*p* < 10^−10^ for each fitness dataset). This may well reflect the greater cellular requirement for proteins in stable complexes, which are more often being captured by affinity methods. For instance, 196 out of 272 translation-related proteins are present in the affinity data, but only 52 out of 272 of these proteins are present in the two-hybrid data. It is then relevant to ask whether this biased sampling might affect conclusions. To this end, we consider only those proteins from the LC set that are found in both the yeast two-hybrid and affinity capture assays. The negative correlation between connectivity and fitness remains robust (Table S1B). Indeed, in this instance the correlation increases from an *r^2^* of 1%–2% to approximately 10%.

All the above results suggest that the more connected a protein might be, the greater the impact on fitness when deleted, assuming the knockout can grow. However, to this conclusion we add one note of caution. We can ask about two sub datasets of the LC set: those being the proteins found only by the yeast two-hybrid method and those found only by the affinity purification method. Surprisingly, in both there is a lack of correlation (Table S1A). One possible reason for the appearance of correlation upon merging of the two types of data is that the mean fitness and mean connectivity of the two sets of data are different. As noted above, the mean fitness of knockouts for proteins in the yeast two-hybrid data was on average higher than for proteins in affinity purification data. Likewise, for proteins only in yeast two-hybrid set, the mean connectivity is 2.16, while for proteins only in the affinity set the mean is 5.2 (*p* < 0.0001, Mann-Whitney U test). Hence, we have one cluster of proteins of high average fitness and low average connectivity and another of lower average fitness and higher connectivity. Merging two such datasets would lead to a negative correlation while none need be seen in either sub datasets. To ask whether the correlation is then real or an artifact of merging potentially biased data, we analyzed a high confidence set of interactions based on the LC set but requiring multi-validation. For those proteins found in the affinity-derived set alone in the high confidence set, we again find a robust negative correlation (*r* = −0.24 and *p* < 0.001). We suggest therefore that the effect is real but easily lost when the data are noisy.

### Essential Genes Are More Highly Connected

As previously reported [13] in both the HTP and the LC datasets, essential genes have on average more partners, i.e., higher connectivity (Mann-Whitney U test comparing essential and nonessential: LC-PI data mean natural log connectivity essential = 1.945 ± 0.03, for nonessentials mean = 1.062 ± 0.02, *p* < 0.0001; for HTP-PI data, mean essentials = 1.55 ± 0.04, for nonessentials, mean = 0.96 ± 0.02, *p* < 0.0001). This is also true if we analyze singleton genes alone (Mann Whitney U test, *p* < 0.0001 for both datasets). Removal of ribosomal proteins does not alter these conclusions (unpublished data). Controlling for protein abundance, the essential genes have higher connectivity than nonessential genes (ANCOVA of log abundance versus natural log of connectivity between essentials and nonessentials, for LC-PI set, *F* = 40.7, *p* < 0.0001, for HTP-PI set, *F* = 14.4, *p* = 0.002).

Curiously, if we restrict analysis to proteins found just in the yeast two-hybrid analyses, there is only a weak tendency for there to be a difference between the essentials and the nonessentials in their connectivity (Essentials mean connectivity: 2.333 ± 0.267, *n* = 42; Nonessentials mean connectivity: 2.12 ± 0.096, *n* = 573; Mann Whitney U test, *p* = 0.056). In contrast, in the proteins identified by the affinity capture methods alone, the result remains highly robust (essentials mean connectivity: 7.85 ± 0.554, *n* = 301, nonessentials mean connectivity: 3.65 ± 0.18, *n* = 754; Mann Whitney U test, *p* < 0.0001). Control for abundance or expression measured by CAI does not alter the later conclusion (ANCOVA: *p* < 0.0001). This suggests that the inability of Coloumb et al. [14] to detect an effect was a consequence of employing yeast two-hybrid data. This would make sense as yeast two-hybrid method is likely to miss many true interactions and so underestimate the number of interactants of the more highly connected proteins. As, moreover, the difference between essentials and nonessentials is seen in all other datasets, we suggest that the connectivity effect is real and is not owing to covariance with expression rate.

### Hubs Do Not Evolve Slower than Non-Hubs

The claim that highly connected proteins evolve slower than others could be in large part an artifact of HTP datasets [15], as these typically report more interactions for proteins that are also highly expressed [11]; moreover, expression rate is a robust predictor of rates of evolution [17]. As highly connected proteins are more likely to be essentials and essentials may evolve slower than nonessentials, we therefore analyze the essential and nonessential genes separately so as to account for any differences between these two in mean rate of protein evolution. One reason many proteins may be nonessential is because they have a duplicate gene (or genes) present in the same genome. As duplicated genes may themselves have unusual rates of evolution [28] (possibly owing to relaxation of functional constraints or to positive selection promoting diversification), it is most valuable to ask whether any of the above results are robust to analysis of singleton genes. For analysis of both duplicates and the dataset en mass see Figures S1 and S2.

**Figure 2:**
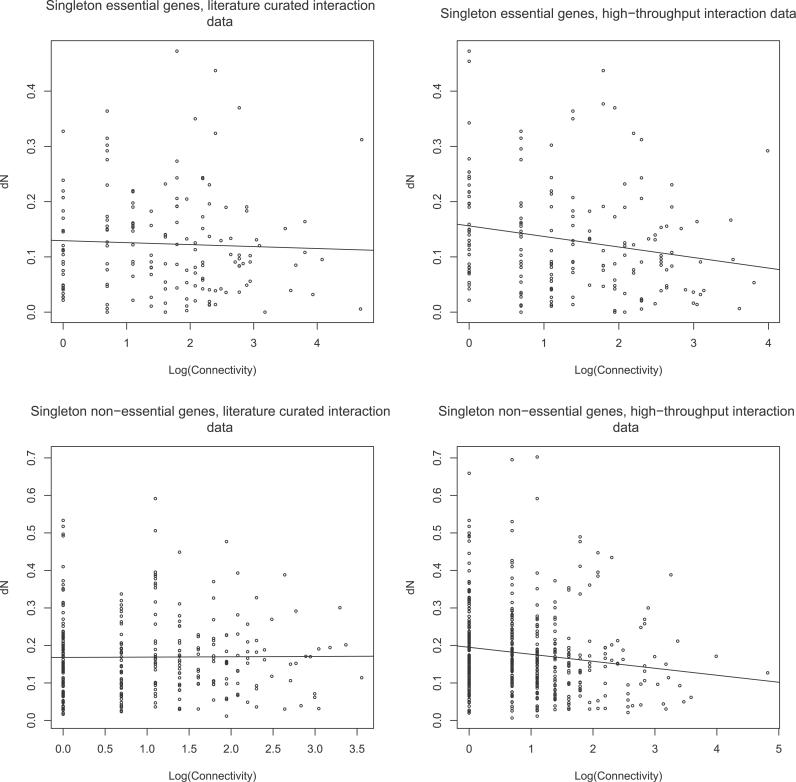
Relationship between Connectivity and Evolutionary Rate Scatter plots of natural log of connectivity and evolutionary rate of S. cerevisiae proteins. Shown here are the plots for singleton genes. For analysis of all genes and those with duplicates see Figures S1 and S2.

Of 3,289 proteins in the LC-PI dataset, evolutionary rate data, i.e., rate of non-synonymous substitution per site, were obtained for 306 singleton nonessentials. Spearman rank correlation, a non-parametric measure that is robust to outliers, between the connectivity and evolutionary rate for these proteins, was not significant (*r =* 0.03*, p* = 0.58) (Figure 2 and Table 2A). The HTP-PI network had 4,474 proteins; evolutionary rate data were obtained for 469 singleton nonessentials (Table 2A). As originally claimed [3,4], a significant negative correlation between the connectivity and evolutionary rate was observed even after accounting for expression level (Table 2A and Figure 2). Because large fractions of protein complexes are essential, restriction of our analysis to nonessentials weakens but does not eliminate the previous correlation between connectivity and evolutionary rate in the HTP-PI network. Similarly, the other two HTP-based networks (Table 2A), including the recent data by Gavin et al. [29] (for both full and a reduced version which controlled for false- positives due to sticky proteins by removing preys that occurred in more than 100 purifications), and another high confidence network, called filtered yeast interactome (FYI), generated by intersecting interactions from various sources [30], showed negative correlation before accounting for expression level. However, in the Gavin (2006) dataset, control for expression level, measured either as protein abundance or by the CAI, removed any significance. The same lack of significance in Gavin (2006) data, after control for abundance, and in the LC data, before and after covariate control, is also seen for singleton essential genes (Table 2B). To account for potential biases in the LC data we repeated the analysis on the LC data without ribosomal proteins and on the subset of LC data that were either two-hybrid based–interactions or affinity purification-based interactions. For all three cases, the null hypothesis of no correlation could not be rejected (Table S2). The lack of correlation is robust to use of Ka/Ks rather than just the protein evolutionary rate (Table S3).

**Table 2:**
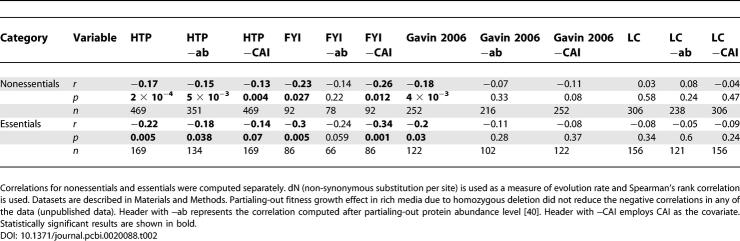
Correlation between Connectivity and Evolutionary Rates

The HTP-PI network may exhibit a negative correlation between connectivity and evolutionary rate because HTP methods preferentially detect interactions of abundant proteins [15,19] that evolve slower than lowly expressed genes [17]. Network data, such as the FYI network (generated by taking intersections of various datasets), while a valid means to enrich for real interactions, further bias HTP data toward highly abundant proteins because interactions between such proteins are more likely to be replicated in HTP datasets. In contrast, interactions that are well established in the primary literature are often not re-published per se, and are therefore not validated in a formal sense, despite being highly controlled and reliable. In addition, HTP approaches tend to recover interactions among proteins that are part of multi-protein complexes, which also evolve more slowly [31]. Indeed, 74% of HTP-PI proteins, for which evolutionary rate data are available, are part of a dedicated protein complex, compared to 64% of LC-PI proteins. The observed negative correlation in HTP datasets also derives from the fact that the average connectivity of subunits of protein complexes is almost two times higher than that of other proteins, in both the LC-PI and HTP-PI networks (*p* < 10^−100^, Spearman).

In conclusion, while we recover the prior strong negative correlation between connectivity and evolutionary rate in various HTP datasets, we find no such correlation in the LC dataset. We conclude that the prior claim that hub proteins are intrinsically slower evolving derives from the nature of the datasets used. This conclusion, however, raises a further problem, namely why do not all highly connected proteins have lower rates of evolution? It is known that sites of non-temporary interaction between proteins are slow evolving [2]. However, do more highly connected proteins necessarily have more such sites or more importantly, a higher density of such sites? To address this issue we obtained the identity of binding residues of yeast proteins for which structures have been solved from the PRISM database [32,33] (see Materials and Methods). Against expectations, the correlation between connectivity and fraction residues associated with binding is negative, albeit only weakly so (for LC data *r =* −0.46, *p =* 0.02 and for HTP data *r =* −0.3, *p =* 0.02; both Spearman rank correlation). This suggests that the underlying assumption that highly connected proteins have a higher density of binding sites does not hold and could explain why hub proteins evolve no faster than average. In part, the negative correlation arises from the fact that proteins with higher connectivity also are longer, although this effect is weak (Spearman rank correlation between protein length and connectivity: for LC-PI data *r* = 0.07, *p* < 0.0001, *n* = 3,256; for HTP-PI data *r* = 0.066, *p* < 0.0001, *n* = 4,142).

### Activity and Lifetime of Protein Hubs Are Under Tight Regulation

The LC-PI network exhibits a highly interconnected topology that links a large fraction of the proteome. To minimize potentially hazardous cross talk, temporal control on the abundance and activity of hubs may be needed to ensure continuous equilibration with binding partners and curb excessive flux through certain pathways. Consistent with this expectation, we find that mRNAs of highly connected proteins have shorter half-lives (*r* = −0.2, *p* = 1 × 10^−22^, Spearman). This negative correlation is true even after partialing-out abundance (*r* = −0.17, *p* = 3 × 10^−10^, Spearman) (abundance is negatively correlated with half-life) and even when considering only nonessentials (*r* = −0.14, *p* = 2 × 10^−11^, Spearman) (essentials have shorter half-life than nonessentials). As a proxy for protein regulation at a post-translational level, we analyzed the phosphorylation status of highly connected proteins. Phosphorylation often dictates regulation through protein interactions and/or protein degradation by the ubiquitin-proteasome system [34,35]. We find that more connected proteins are more likely to be phosphorylated (*r* = 0.06, *p* = 9 × 10^−4^, Spearman). Thus, one trade-off for broad specificity [36] may be the energetic cost of “just-in-time” synthesis, which helps prevent entrainment of hubs by a select set of interaction partners.

## Discussion

Analysis of LC-PI data suggests that prior analyses of the relationship between connectivity and rate of evolution were a product of the biases inherent in HTP data. In contrast, the relationship between dispensability and connectivity seems more robust. In particular, the claim that more highly connected proteins tend to be more likely to be essential is reported in all datasets, although this is only a trend when the data are derived exclusively from yeast two-hybrid assays. Similarly, the yeast two-hybrid data fail to support the idea that within the class of nonessential genes there is a correlation between growth rate and connectivity. Otherwise, we find reasonable support for this possibility, although in some instances the correlation is weak.

That highly connected proteins do not evolve slowly is intriguing given the simple intuition that the two attributes should covary. However, the logic of this intuition relies on the two interrelated assumptions: that sites of mutual protein–protein binding should be slow evolving and that proteins with numerous interactions should have a higher density of binding sites as they have more partners. A third implicit assumption is that the proportion of sequence defined by binding sites is large enough to impact on rates of evolution given high variation in rates of evolution outside of pairing sites. The first assumption is partially upheld: sites of non-temporary binding are indeed slow evolving [2]. Given this, why are not more highly connected proteins slower evolving? A key alternative possibility is that highly connected proteins tend to re-use the same binding site when interacting with multiple different proteins. Our analysis of well-described binding domains suggests this to be so and moreover, that genes with multiple interactants tend to be longer proteins; hence, for a given number of binding sites, the density of the sites will be lower in the longer proteins. However, the data for estimating the density of sites are limited and may also be biased, most especially toward proteins in obligate complexes. Nonetheless, the unexpected lack of a positive correlation between proportion of sequence involved in binding and connectivity provides evidence to support the conclusion that connectivity is not related to rate of protein evolution.

The finding that hub proteins tend to be encoded by mRNAs with rapid turnover rates provides an alternative explanation for the lack of correlation between connectivity and rate of evolution. From our data it would appear that many hub proteins are adapted to rapid turnover and/or regulation as they have both short half-lives and more phosphorylation sites. The short half-life in particular suggests that many hubs are not part of long-term stable interactions and instead form dynamic complexes that are readily removed from the system (or inactivated by phosphorylation) once a given task at a given time is performed. Many dynamic interactions likely occur via weak interactions at less conserved and hence less constrained binding surfaces, such as in the instance of kinase-substrate interactions. However, we note that dynamic interactions can also be mediated via conserved binding pockets that bury large surface areas of the interacting partners. In the latter instance, subunits marked for rapid turnover by phosphorylation, which often directs ubiquitin conjugation and subsequent degradation, can be readily stripped from the rest of the complex by the 26S proteasome and rapidly degraded [37,38]. In this manner, the same dedicated binding site may be used to link to many different interaction partners. A definitive test of the “just-in-time” attribute of hub interactions will require measurement of protein half-lives on a proteome-wide scale and systematic determination of the structural basis for transient protein interactions.

## Materials and Methods

### Interaction networks.

The HTP dataset was created by union of four HTP studies [21–24]. The LC-PI dataset [13] was obtained from the BioGRID database (http://www.thebiogrid.org), and the FYI dataset was obtained from Han et al. [30]. A dataset corresponding to ~2,000 purified stable yeast protein complexes was created by connecting each bait to its prey in a spoke model [29]. This latter analysis was also repeated with a reduced dataset that controlled for promiscuous interactions by removing preys that occurred in more than 100 purifications. Descriptive statistics of these networks are shown in Table 3.

**Table 3:**
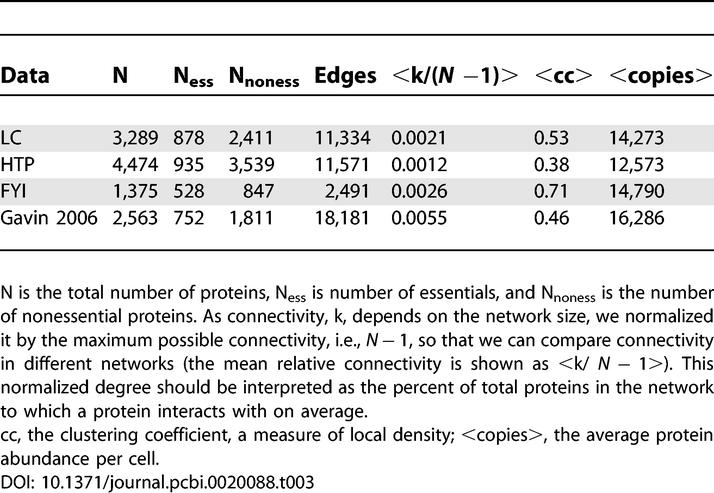
Descriptive Statistics of Data Used in the Analysis

### Miscellaneous data.

Evolutionary rate data (non-synonymous substitution per site and synonymous substitution per site) for the four-way *stricto sensu* species alignments for 3,036 genes [39], proteome-wide protein abundance [40], and genome-wide yeast mRNA half-life [41] datasets were as described. CAI values were calculated as described [27] using 30 most highly expressed yeast genes [42]. Predicted phosphorylation data were obtained from Scansite database at: http://scansite.mit.edu [43] using a high stringency cut-off. Scansite identifies short protein sequence motifs that are recognized by modular signaling domains that are phosphorylated by protein Ser/Thr- or Tyr-kinases, or that mediate specific interactions with protein or phospholipid ligands. GO component complex membership information was obtained from the SGD database (http://www.yeastgenome.org) [44]. To identify singleton genes, a yeast versus yeast BLASTP was done with E-value threshold of 0.1 and percent identity cut-off of 20; those proteins which did not return a hit using these parameters were considered to be singletons. This procedure resulted in 1,558 singletons. Proteins that were part of the “core” minus the “attachments” [29] were considered to be stable complex subunits. Homozygous single-gene deletion fitness data in rich media (YPD) were as described [45,46]. Average of the two-replicate growth rate fitness measure of the homozygous deletion in rich media (YPD) was taken from the latter dataset.

### Protein interaction residues.

Identity of binding residues data was obtained from the PRISM database [32]. Identity of residues taking part in protein–protein interactions were predicted using a computational approach that used structure information (mostly on multi-protein complexes) from the Protein Data Bank (PDB) [33]. Briefly, interfaces were defined as the set of residues that made non-covalent contacts with residues on other chains or those that were in the vicinity of these contacting residues. Two residues from the opposite chains were marked as interacting if there was at least a pair of atoms, one from each residue, at a distance smaller than the sum of their van der Waals radii. Of the 166 S. cerevisiae proteins, for which interaction residue information was available, only those proteins that had a unique yeast ORF name were retained. Number of binding residues in the pair-wise domain interfaces was normalized by protein length to obtain the fraction of the sequence involved in binding. For all data used in the analysis see Dataset S1.

### Statistical methods.

The partial Spearman correlation between two variables, controlling for a third variable, was computed using the standard formula [26]

## Supporting information

Dataset S1 Data Used in This Analysis Columns: Gene, ESS? (Essential genes, 1 nonessential 0), y2h_or_aff (method used to find interaction in LC data, 1 = y2h only, 2 = affinity only, 3 = both, 0 = neither), k_lc (number of interactants in LC dataset, k_htp (number of interactants in HTP dataset), Abd (protein abundance), CAI, Rib (ribosomal, 1 = yes, 0 = no), fr_bind (fraction of residues involved in binding), mRNA_1/2 (mRNA half-life), phosphorylation (number of phosphorylation sites).
(980 KB XLS)

Dataset S2 Top 10% of Genes by Connectivity in the LC Dataset (55 KB TXT)

Dataset S3 Top 10% of Genes by Connectivity in the HTP Dataset (50 KB TXT)

Figure S1 The Relationship between Natural Log of Connectivity and Rate of Protein Evolution for All Genes NB the slopes on the regression lines are not significantly different from zero for both LC datasets, but are highly significant (*p* < 0.0001) for the HTP data. For the essential genes in the LC set the non-parametric correlation is weakly significant (*r* = −0.12; *p* < 0.01) but sensitive to control for protein abundance.
(239 KB PDF)

Figure S2 The Relationship between Natural Log of Connectivity and Rate of Protein Evolution for Genes with Duplicates NB the slopes on the regression lines are not significantly different from zero for both LC datasets, but are highly significant for the HTP data. For the essential genes in the LC set the non-parameteric correlation is weakly significant (*r* = −0.12; *p* < 0.05) but sensitive to control for protein abundance.
(141 KB PDF)

Table S1 Degree versus Fitness for Subsets of LC data (1A) and Degree versus Fitness for Proteins Found in Both Y2h and Affinity Purification Methods (1B) (28 KB DOC)

Table S2 Degree versus Evolutionary Rate for Subsets of LC Data (Using dN) (25 KB DOC)

Table S3 Degree versus Evolutionary Rate for Subsets of LC Data (Using dN/dS) (25 KB DOC)

## Abbreviations

CAI: codon adaption index
FYI: filtered yeast interactome
HTP-PI: high-throughput protein interaction
LC-PI: literature-curated protein interaction
