# Supplementary material for: Evolutionary and Physiological Importance of Hub Proteins
Source: PLoS Comput Biol. 2006 Jul 14;2(7):e88. doi: 10.1371/journal.pcbi.0020088 (PMC1500817; doi:10.1371/journal.pcbi.0020088)
Supplement: Supplementary file 1 [file pcbi.0020088.sg001.pdf]

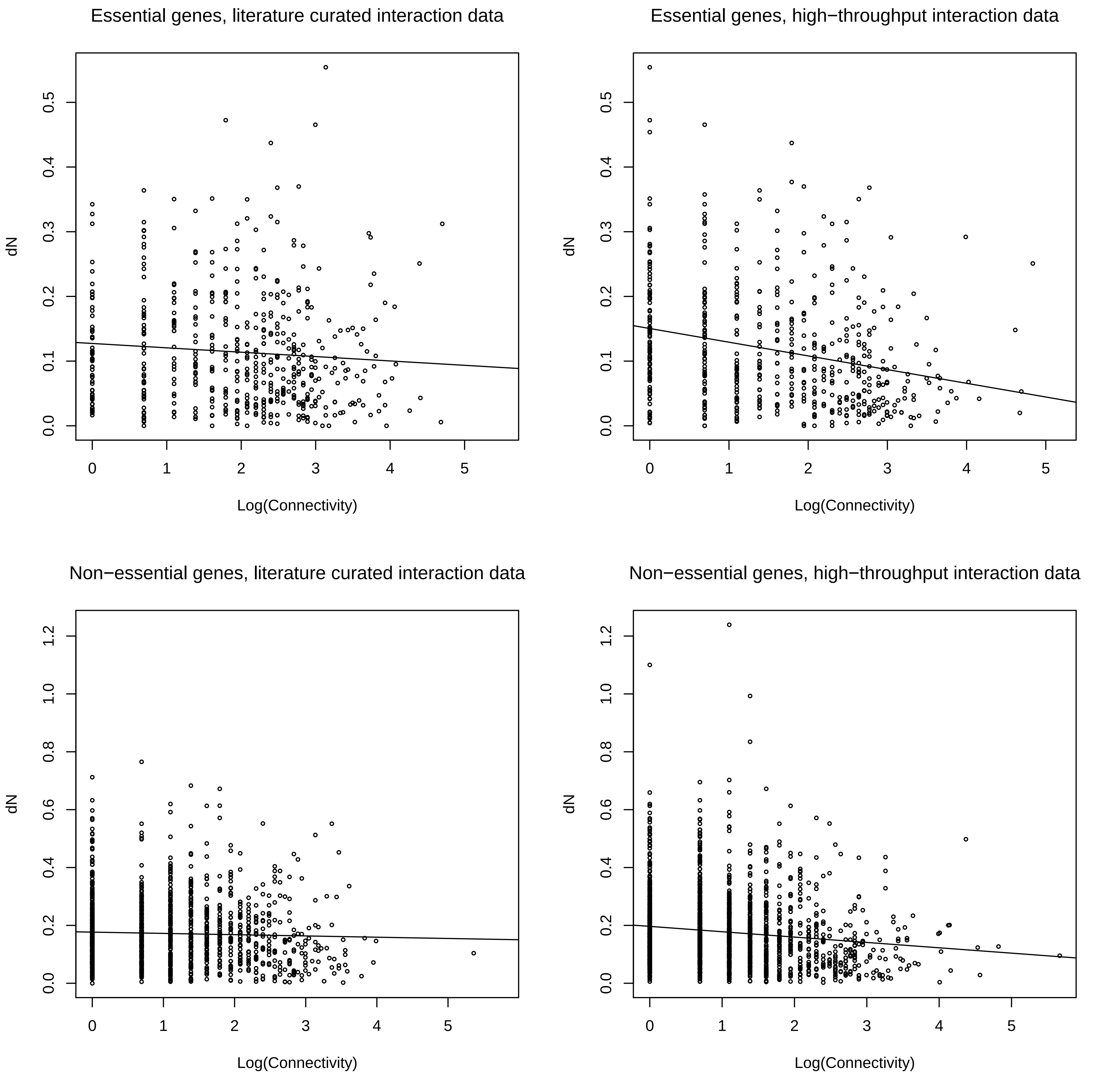
Essential genes, literature curated interaction data
Essential genes, high−throughput interaction data
Non−essential genes, literature curated interaction data
Non−essential genes, high−throughput interaction data
dN
Log(Connectivity)
0.0
0.1
0.2
0.3
0.4
0.5
0.6
0.8
1.0
1.2
0
1
2
3
4
5
