# Supplementary material for: Evolutionary and Physiological Importance of Hub Proteins
Source: PLoS Comput Biol. 2006 Jul 14;2(7):e88. doi: 10.1371/journal.pcbi.0020088 (PMC1500817; doi:10.1371/journal.pcbi.0020088)
Supplement: Supplementary file 2 [file pcbi.0020088.sg002.pdf]

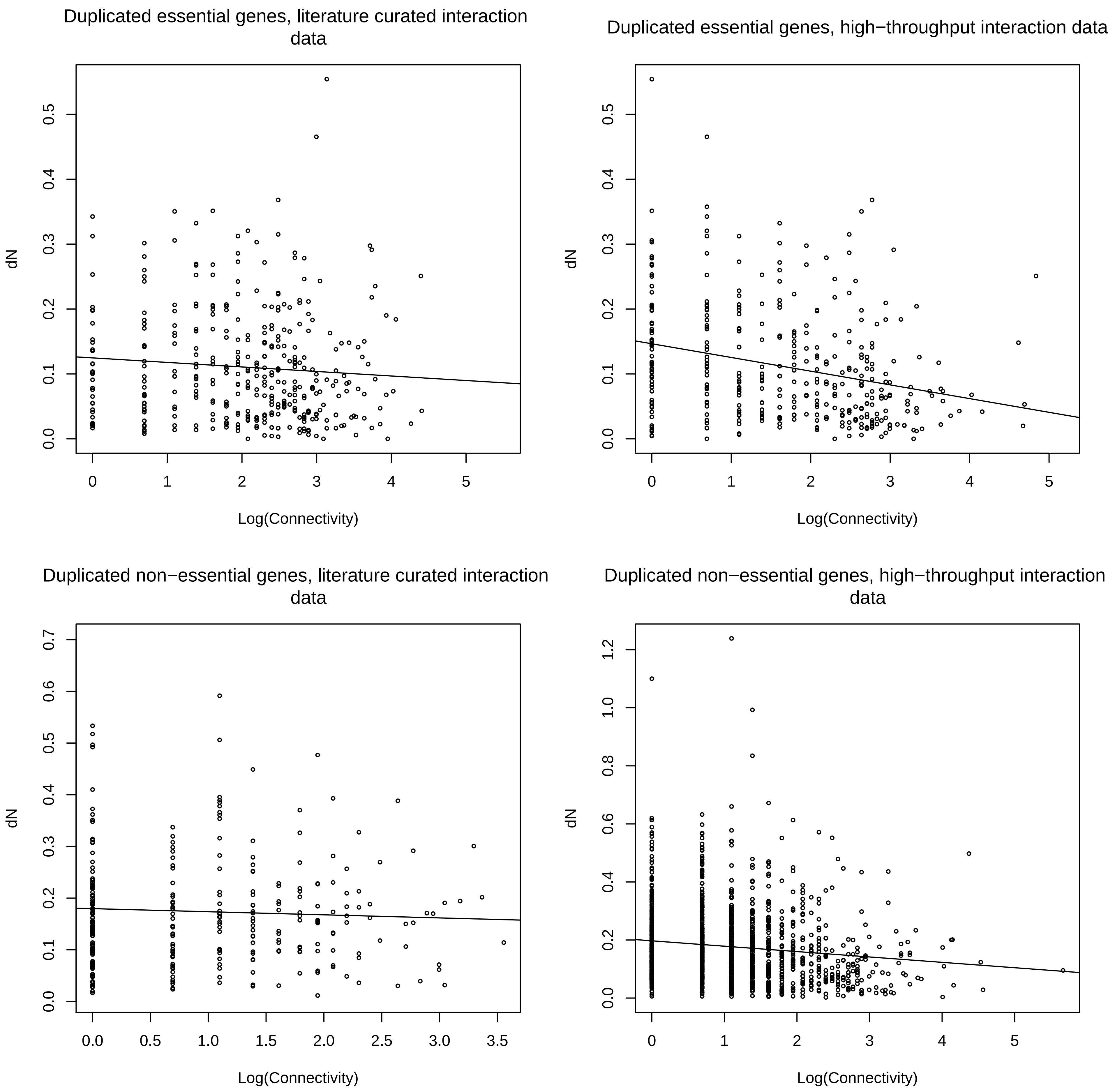
Duplicated essential genes, literature curated interaction data
Duplicated essential genes, high−throughput interaction data
Duplicated non−essential genes, literature curated interaction data
Duplicated non−essential genes, high−throughput interaction data
dN
Log(Connectivity)
0.0
0.1
0.2
0.3
0.4
0.5
0.6
0.7
0.8
1.0
1.2
0
1
2
3
4
5
0.0
0.5
1.0
1.5
2.0
2.5
3.0
3.5
