# Supplementary material for: Evolutionary and Physiological Importance of Hub Proteins
Source: PLoS Comput Biol. 2006 Jul 14;2(7):e88. doi: 10.1371/journal.pcbi.0020088 (PMC1500817; doi:10.1371/journal.pcbi.0020088)
Supplement: Supplementary file 3 [file pcbi.0020088.st001.doc]

### Supplementary Table 1a: Degree vs Fitness for subsets of LC data

Nr = LC without ribosomes, Y2h= LC interactions from two hybrid experiments only, Aff=LC interactions from affinity purification methods only. Fitness data type: steinmetz §, deutschbauer ¶

|  | Nr^§^ | Nr-ab^§^ | Nr-cai^§^ | Nr^¶^ | Nr-ab^¶^ | Nr-cai^¶^ | Y2h^§^ | Y2h-ab^§^ | Y2h-cai^§^ | Y2h^¶^ | Y2h-ab^¶^ | Y2h-cai^¶^ | Aff^§^ | Aff-ab^§^ | Aff-cai^§^ | Aff^¶^ | Aff-ab^¶^ | Aff-cai^¶^ |
| --- | --- | --- | --- | --- | --- | --- | --- | --- | --- | --- | --- | --- | --- | --- | --- | --- | --- | --- |
| r | -0.24 | -0.22 | -0.26 | -0.25 | -0.23 | -0.27 | -0.04 | -0.07 | -0.07 | -0.08 | -0.07 | -0.1 | -0.09 | -0.08 | -0.09 | -0.11 | -0.1 | -0.11 |
| p | 8e-7 | 1e-4 | 7e-8 | 2e-7 | 4e-5 | 2e-8 | 0.5 | 0.4 | 0.3 | 0.2 | 0.3 | 0.1 | 0.1 | 0.2 | 0.1 | 0.04 | 0.1 | 0.06 |
| N | 412 | 311 | 412 | 427 | 323 | 427 | 250 | 197 | 250 | 256 | 201 | 256 | 297 | 247 | 297 | 309 | 257 | 309 |

### Supplementary Table 1b: Degree vs Fitness for proteins found in both Y2h and affinity purification methods

Ov = overlap set, singlton proteins only, Fitness data type: steinmetz §, deutschbauer ¶

|  | Ov^§^ | Ov-ab^§^ | Ov-cai^§^ | Ov^¶^ | Ov-ab^¶^ | Ov-cai^¶^ |
| --- | --- | --- | --- | --- | --- | --- |
| r | -0.32 | -0.28 | -0.33 | -0.32 | -0.25 | -0.32 |
| p | 7e-5 | 0.002 | 5e-5 | 9e-5 | 0.005 | 8e-5 |
| N | 144 | 128 | 144 | 149 | 132 | 149 |
