# Supplementary material for: Evolutionary and Physiological Importance of Hub Proteins
Source: PLoS Comput Biol. 2006 Jul 14;2(7):e88. doi: 10.1371/journal.pcbi.0020088 (PMC1500817; doi:10.1371/journal.pcbi.0020088)
Supplement: Supplementary file 4 [file pcbi.0020088.st002.doc]

### Supplementary Table 2: Degree vs Evolutionary rate for subsets of LC data (using dN)

data type: essentials §, non-essentials ¶

|  | Nr^§^ | Nr-ab^§^ | Nr-cai^§^ | Nr^¶^ | Nr-ab^¶^ | Nr-cai^¶^ | Y2h^§^ | Y2h-ab^§^ | Y2h-cai^§^ | Y2h^¶^ | Y2h-ab^¶^ | Y2h-cai^¶^ | Aff^§^ | Aff-ab^§^ | Aff-cai^§^ | Aff^¶^ | Aff-ab^¶^ | Aff-cai^¶^ |
| --- | --- | --- | --- | --- | --- | --- | --- | --- | --- | --- | --- | --- | --- | --- | --- | --- | --- | --- |
| r | 0.04 | 0.11 | -0.07 | -0.1 | -0.06 | -0.11 | 0.03 | 0.04 | -0.05 | -0.08 | 0.025 | -0.06 | 0.03 | 0.03 | -0.02 | -0.01 | 0.05 | -0.01 |
| p | 0.55 | 0.11 | 0.27 | 0.23 | 0.52 | 0.19 | 0.71 | 0.63 | 0.54 | 0.48 | 0.84 | 0.56 | 0.68 | 0.69 | 0.78 | 0.88 | 0.61 | 0.88 |
| N | 278 | 213 | 278 | 148 | 115 | 148 | 167 | 131 | 167 | 88 | 70 | 88 | 200 | 169 | 200 | 143 | 110 | 143 |
